# Fukuoka: Adapting to climate change through urban green space and the built environment?

**DOI:** 10.1016/j.cities.2019.05.007

**Published:** 2019-10

**Authors:** Leslie Mabon, Kayoko Kondo, Hiroyuki Kanekiyo, Yuriko Hayabuchi, Asako Yamaguchi

**Affiliations:** a School of Applied Social Studies, Robert Gordon University, Scotland, United Kingdom; b Faculty of Design, Kyushu University, Japan; c Global Innovation Center, Kyushu University, Japan

**Keywords:** Climate change adaptation, Fukuoka, Urban climate change governance, Urban greenspace, Urban planning

## Abstract

This paper profiles Fukuoka City in Kyushu, Japan. We focus on the city's local climate change adaptation policies, and in particular the role of urban and greenspace planning in facilitating adaptation actions within Fukuoka. Fukuoka is a humid subtropical city which is currently experiencing significant population and economic growth. It has also made comparatively rigorous advances in climate adaptation, in a country context where local governments have been criticised for focusing more on mitigation. Fukuoka hence may yield lessons for other rapidly urbanising subtropical Asian cities. We illustrate that Fukuoka has a long tradition of science-policy connection towards the creation of a liveable urban environment. This creates a favourable research and policy infrastructure for adaptation, in particular mitigation of heat risk. This is evidenced in consideration of climate issues within the city's greenspace plans since the 1990s, and in an extensive body of underpinning applied research from local institutions into urban thermal environments in particular. Fukuoka's green terraced ACROS building has come to symbolise adaptation via the built environment, and has been followed by the emergence of further green roofs and through citizen and private sector involvement in smaller-scale greening actions. We caution that challenges remain around connecting different sections of local governments, and in maintaining climate and environmental imperatives in the face of ongoing development and expansion pressures.

## Highlights

• Fukuoka among early adopters of municipal climate change adaptation action in Japan
• Fukuoka demonstrates adaptation responses for an expanding sub-tropical Asian city.
• Understanding of wind corridors and cooling by greening informs Fukuoka city plans.
• Adaptation via planning continues much longer trend of liveable urban environment.
• Challenges remain in balancing economic development pressures with climate actions.

## 1 Introduction and rationale

Although Japanese policy on climate change has been criticised for focusing more on mitigation than adaptation at the local^1^ level (Baba et al., 2017; Kameyama, 2016), Fukuoka City has considered adaptation to the effects of environmental change within its greenspace and urban plans since the late 1990s (Fukuoka City, 1999). Following the adoption of the Kyoto Protocol in 1997, local governments in Japan have engaged enthusiastically with climate change mitigation via climate change plans geared towards emissions reduction and renewable energy deployment (Kameyama, 2016). However, progress on adaptation has been much slower. The 2018 Climate Change Adaptation Act now mandates municipal governments to form Local Climate Change Action Plans. Yet up until this point, few Japanese cities have had specific laws and plans to address adaptation action, and there is limited evidence of practical adaptation actions at the local level (Hijioka et al., 2016). In Fukuoka, by contrast, locally-based researchers have undertaken applied research on the relationship between urban greening, the built environment and urban thermal environments for several decades (e.g. Katayama et al., 1991; Nitta, Azuma, & Ishii, 1981; Yoda & Katayama, 1998). Fukuoka City Government also released in 2016 a climate change countermeasures action plan to integrate climate and environmental plans and link mitigation with adaptation, continuing actions which have developed since the city made its first local climate plan in 1994 (Fukuoka City, 2016a). This initial formalised climate action came several years ahead of comparable western Japanese cities such as Kyoto (1997); Kobe (2000); Osaka (2002); and Hiroshima (2003).

Fukuoka is therefore significant for several reasons. Firstly, it can be considered a front-running city within Japan for local climate change policy, and also for actions targeted specifically towards adaptation to environmental change supported by the applied research history outlined above. Second, despite national trends, the population in Fukuoka is growing at the fastest rate of any Japanese city (Fukuoka Asian Urban Research Center, 2018), with resulting urban development and expansion (Fukuoka City, 2018a). Third, Fukuoka is among the southernmost designated cities in Japan in the humid subtropical climate zone. Assessing how Fukuoka has integrated climate adaptation-related issues into its local policies despite slow national progress, and clarifying remaining challenges, may hence yield lessons for other expanding Asian subtropical cities facing increasing climate-related risks such as urban heat island effects and flooding. In the wider international context, evaluation of how Fukuoka has managed urban heat in particular can contribute to a knowledge gap identified by the IPCC's Cities and Climate theme around knowledge of the relation between planning and the thermal environment in non-European or North American contexts (Prieur-Richard et al., 2018). Elaboration of how Fukuoka has drawn on techno-scientific expertise and evidence to support adaptation via urban planning can feed into debates – as per a recent *Nature Sustainability* expert panel - on how ‘the science of cities’ can inform urban climate responses (Acuto et al., 2018).

For the purpose of this City Profile, we focus on Fukuoka City as an administrative unit. This is because (a) Fukuoka City Government is the municipal administrative unit governing the built-up area of Fukuoka City, and hence can make finer-scale recommendations than the higher-level prefectural government; and (b) Fukuoka Prefecture in any case is required to develop adaptation actions for a mixed rural and urban context. Indeed, it is noted in Fukuoka City's climate plan that the prefectural climate plan provides general overarching guidance for the city's actions, but that these are translated into practice via policies and spatial plans produced by the city government (Fukuoka City, 2016a). Unless otherwise stated, we therefore use *Fukuoka City* to describe specific actions undertaken by Fukuoka City Government, and *Fukuoka* to refer to more general social and cultural characteristics in the wider Fukuoka area. Actions and policies relevant to Fukuoka City but enacted by Fukuoka Prefecture are of course considered within the profile as and when appropriate.

## 2 Data sources

The information presented in this paper is derived from three sources. First is policy documentation produced by Fukuoka City and also Fukuoka Prefecture relating to climate change adaptation and the urban environment, including climate change plans, greenspace plans, and urban planning masterplans. Second is a review of scholarly research outputs pertaining to urban planning and environmental change with an empirical focus on Fukuoka. These were collated via ‘snowball’ sampling based on (a) searching Web of Science for the terms ‘Fukuoka’ and ‘climate’, ‘greenspace’ or ‘planning’; and (b) searching the Japan Society for the Promotion of Science's KAKEN database for additional Japanese-language projects and outputs, using Japanese equivalents of the same search terms. Third are eight in-depth interviews with people associated with local adaptation responses: one at Fukuoka City Environment Division; one at the Fukuoka City Green City Promotion Department; one at Fukuoka Prefecture Environment Division; one at the Fukuoka Center for Climate Change Actions; one at Kyushu Environmental Evaluation Association; one with an academic involved in the expert committee for Fukuoka City's Climate Change Countermeasures Action Plan; one with an academic involved in local and regional adaptation planning in Japan; and one at a private-sector consultancy providing public bodies with advice on climate change responses and environmental management actions. Whilst the profile is based largely on the review of policy and scholarly outputs, the interviews provided additional contextual information. This allowed observations from the policy analysis and review of academic research to be clarified, and also allowed respondents to provide a more critical and evaluative insight on the extent to which policy rhetoric translates into practice (see Section 6.5 in particular).

## 3 Location and social and environmental characteristics

**Fig. 1:**
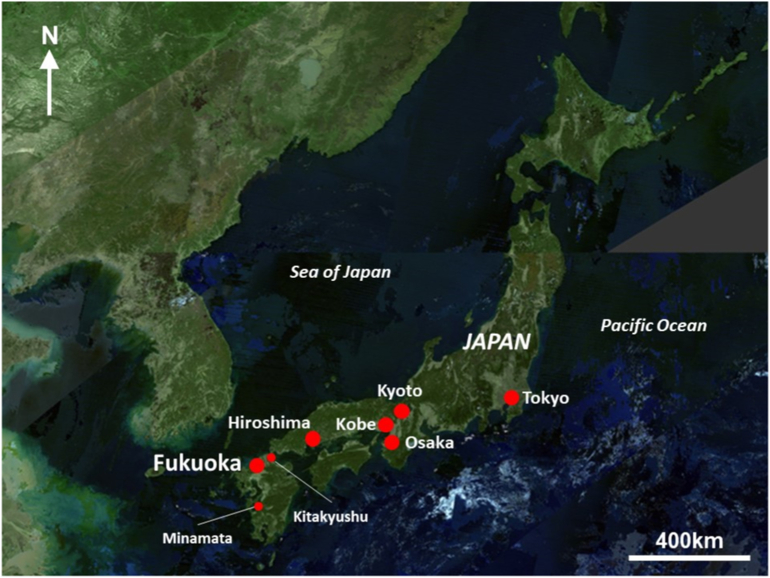
Location of Fukuoka City within Japan, showing major cities and locations mentioned in the city profile.

Fukuoka is located in the north of Kyushu, the southern-most of Japan's four main islands (see Fig. 1). It is bordered by Hakata Bay (which opens into the Genkai Sea) to the north, and by the Sangun Mountain Range to the east and the Sefuri Mountain Range to the south-west. Fukuoka City is located within Fukuoka Prefecture, on the Fukuoka Plain. Fukuoka Prefecture is one of the 47 regional administrative units in Japan, and includes the urbanised area of Kitakyushu and the more rural areas of Chikuho and Chikugo over an area of 4584 km^2^. The population of Fukuoka Prefecture was approximately 5.1 million people in 2018, of whom around 3.8 million people were living in the urbanised areas of Fukuoka and Kitakyushu (Fukuoka Prefecture, 2019).

(Source: adapted from Geospatial Information Authority of Japan, 2019)

The population of Fukuoka City was approximately 1.5 million people in 2018, over an area of 340 km^2^ (Fukuoka City, 2017). Fukuoka City has a humid subtropical climate, with the highest temperatures reaching around 37 °C in the months of July and August and an average of 1612 mm of rainfall annually (see Section 6 for a more comprehensive overview of weather and climate and its relation to climate change) (Fukuoka City, 2017).

Fukuoka's population grew by 7.1% between 2010 and 2017, compared to 5.8% for Tokyo – giving it the biggest growth of any major city in Japan (Fukuoka Asian Urban Research Center, 2018). A large proportion of this population change is due to in-migration, with 6.8% of the population in-migrants from elsewhere in Japan or overseas (Fukuoka Asian Urban Research Center, 2018) Most recent data from 2016 shows that people over 65 – a group often considered vulnerable to climate risks – make up 21.4% of the city population (Fukuoka City, 2017) compared to 27.2% for Japan overall (Ministry of Internal Affairs and Communications, 2017). According to data from 2014, service industries (25.6%) made the biggest contribution to Fukuoka City's real gross regional product by economic activity, followed by wholesale (13.3%) and real estate activity (12.6%) (Fukuoka Asian Urban Research Center, 2018).

## 4 Brief history of development

Fukuoka developed as twin cities from the 17th Century onwards. Fukuoka, to the west of the Naka River, was a castle town of the samurai military class. Hakata, to the east of the river, was a city of merchants. The legacy of this historical structure is still visible today in the divide between the commercial centre of the city east of the Naka River, and the administrative and entertainment areas to its west.

Settlement in the area where Fukuoka is now located began approximately 26,000 years ago, when people crossed from the Asian continent. Around 15,000 years ago, the region became separated from the rest of Asia by what is now the Genkai Sea. Rice farming superseded a hunter-gatherer lifestyle, and the people of the region started to run agricultural communities. The first state in the Japanese archipelago was established in the region, with records showing that in 57 CE, the Emperor of the Late Han Dynasty gave a gold seal to envoys sent by the king to request support for their kingdom (Fukuoka City Museum, 2013).

After the centre of the nation moved east, the region where Fukuoka is located today played an important role for diplomacy, commerce and defence. The government of the time established a diplomatic facility called the Korokan, which in turn was succeeded as a centre of commerce by an area known as *Hakata*, which supported Chinese traders. Following attempted Mongolian invasions in the late 13th Century, trade with Ming (China), the Ryukyu Islands and the Korean Peninsula made Hakata a flourishing trading port (Fukuoka City Museum, 2013). The Hakata name is still visible today as one of the seven wards in Fukuoka City, and as the name for the main high-speed rail station in the city.

In 1601, the House of Kuroda established a new castle and town near to Hakata. This new town was named *Fukuoka* after the feudal lord Nagamasa Kuroda's birthplace in Okayama Prefecture. In April 1889, the government issued an order for municipalisation, and the towns of Fukuoka and Hakata were merged. ‘Fukuoka’ was chosen as the name for the new city. At this time, their populations were 20,410 and 25,677 respectively (Fukuoka City, 2009a).

Fukuoka City continued to expand and developed a streetcar network in the early part of the 20th Century, but the city was heavily damaged by bombing raids in World War II. The city rebuilt and continued to develop along with Japan's post-War economic miracle, becoming Kyushu's core city by the mid-1970s. At this time, the Tenjin Underground Shopping Mall, Tenjin Station on the Fukuoka Municipal Subway Line, and several large department stores opened in the Tenjin area. The opening of the Tenjin Subway Line shifted in the flow of people in the central part of Fukuoka, changing the flow from along Meiji-Dori on an east-west axis, to Watanabe-Dori on a north-south axis (Fukuoka City, 2009b). In 1987, in response to citizen preferences for comfort and aesthetics over function and efficiency, Fukuoka City Government enacted legislation focusing on the urban landscape and initiated the Fukuoka City Urban Landscape Award to recognize those who contributed to the creation of an appealing urban landscape (Fukuoka City, 2009b).

## 5 Urban form, current/future developments, and climate adaptation and greenspace governance

### 5.1 Urban development and governance context

**Fig. 2:**
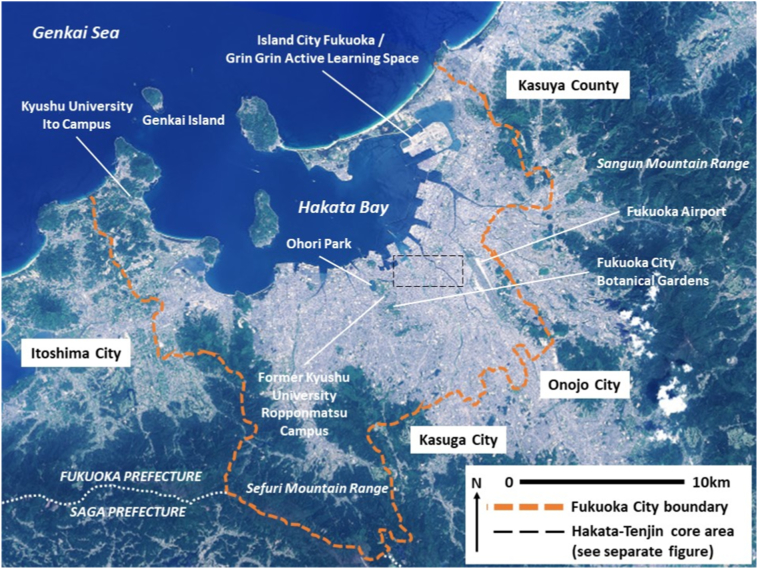
Fukuoka City.

**Fig. 3:**
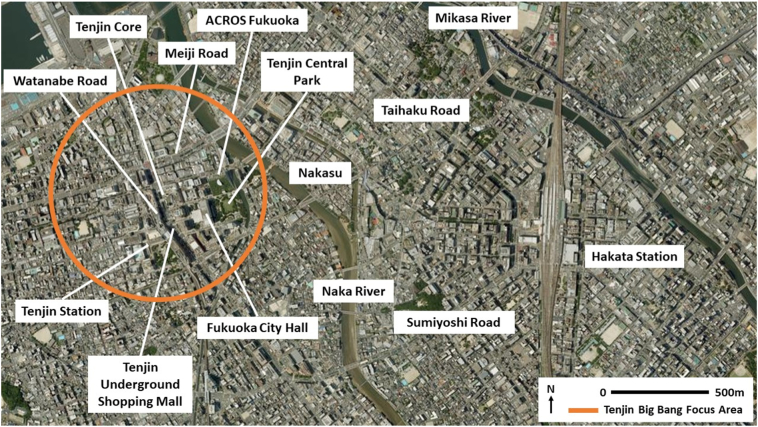
Hakata-Tenjin core area.

Whilst the different characters of east- and west Fukuoka are rooted in several centuries of history, the precise urban form of the city and its current development trajectory are informed by topographical constraints and more recent events (see Fig. 2, Fig. 3). The current layout of the city's central area still largely reflects the form laid down during systematic post-World War II reconstruction planning. Meanwhile, mountains to the south and the sea to the north mean development entails either expansion in an east-west direction, or intensification.

(Source: source: adapted from Geospatial Information Authority of Japan, 2019)

(Source: adapted from Geospatial Information Authority of Japan, 2019)

After Fukuoka was heavily damaged by saturation bombing during World War II, the current form of the city's central area was largely set by implementation of post-War reconstruction plans in the 1950s and 1960s (Fukuoka City, 1979). Hakata Station was moved 600 m to the north at this time, and became the meeting point for the Sumiyoshi and Taihaku roads. With development to the south constrained by mountain ranges, land reclamation has taken place into Hakata Bay to the north. Reclamation commenced in the 1930s, but intensified from the 1970s onwards to the north-east in particular (Ishibashi & Shibata, 2014). Island City Fukuoka, completed in 2005, is one such development with mixed residential, recreational and institutional land uses.

As outlined in Section 3, Fukuoka has experienced notable population growth and in-migration. Yet in addition to geographical constraints, noise protection controls around Fukuoka Airport to the east, and strict municipal urbanisation control regulations to manage sprawl, further limit expansion and development. Current real estate development and expansion is thus focused in the direction of Itoshima City to the west (aided by the relocation of some parts of Kyushu University from the Ropponmatsu area of Jonan Ward to a new campus in the far west of Fukuoka City), and Kasyua County to the north-east. Development has also taken place into the 21st Century along the Japan Railways line to Kasuga City and Onojo City to the south-east, however this area is now reaching saturation. Given these geographical and land use constraints, the current Fukuoka City Urban Planning Masterplan states that consolidation and development of the Hakata-Tenjin core area is key to attaining the goal of making Fukuoka City an interchange and focal point for Asia, and also to extracting more economic value from Fukuoka's core area in line with the city's growth (Fukuoka City, 2014). To aid this process, Fukuoka City Government has initiated the ‘Tenjin Big Bang’ project, which has relaxed regulations on the height of buildings and on floor area ratio to encourage redevelopment of 30 old buildings in Tenjin by 2024 (Fukuoka City, 2018a).

**Fig. 4:**
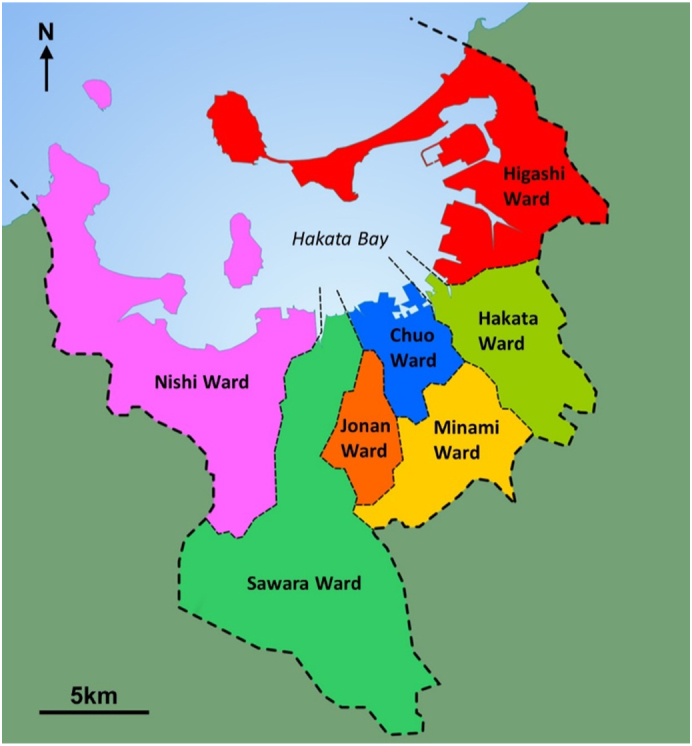
Wards of Fukuoka City.

Administratively, Fukuoka City is divided into seven wards (see Fig. 4). The ward names loosely correspond to names of areas from the time of the Kuroda samurai clan in the 17th Century. However, the wards in their contemporary form are a superimposed political structure. The wards were created when Fukuoka was made a designated city by the Japanese government in 1972. Initially the city consisted of five wards, however the former Nishi Ward was subdivided into Jonan Ward, Nishi Ward and Sawara Ward in 1982. The main business and commerce district is Hakata Ward, whilst most entertainment and retail is located in the Tenjin area of Chuo Ward. Together, the Hakata and Tenjin areas make up the core of Fukuoka City.

(Source: produced by authors)

Most of the Fukuoka metropolitan area falls under the jurisdiction of Fukuoka City Government. This is the municipal government for Fukuoka City, and is located within Fukuoka Prefecture – the regional level of government. As far as climate change governance is concerned, it is Fukuoka City (i.e. the municipal level) which is mandated to produce a climate adaptation plan for the city. The municipal government is responsible for translating climate-related legislation, policies and directives from the national and prefectural governments into practice. Fukuoka City Government is able to respond to these directives through control of areas such as land use/greenspace planning and municipal climate action plans, as discussed in Section 6.

### 5.2 Urban form and climate adaptation context

Adaptation to climate change in Fukuoka City comes against a backdrop of an increasing population, with associated pressures on urban development and awareness of the need to preserve or increase greenspace. The population density of Fukuoka City grew from 3602 people per km^2^ in 1990, to 3907 people per km^2^ in 2000, and 4481 people per km^2^ in 2015 (Statistics Agency of Japan, 2015). Fukuoka City acknowledges a problem in that the ratio of green coverage in Fukuoka City declined from 60.2% in 1985, to 58.0% in 1996, and 55.4% in 2007 (data from the last time the city's greenspace plan was updated) (Fukuoka City, 2009c). Over the same time periods, the ratio of green coverage in the city's defined built-up areas reduced from 25.9% (1985) to 23.8% (1996), then down to 20.7% (2007).

Nonetheless, through actions such as planting street trees, greening of public spaces, and greening of private residences, the area of ‘created greenery’ in Fukuoka City's defined built-up areas was increased by 9.1% from 1996 to 2008, and over the same time period the area of formal parks and greenspaces increased by 21.4% due to creation and incorporation of new parks (Fukuoka City, 2009c). Table 1 below shows how this greenery and greenspace is distributed by ward. Moreover, as actions such as rooftop gardens, green walls and proliferation of greenery across the city are stated as important heat island mitigation actions in the city's greenspace and urban plans, the number of community and private sector greening actions (e.g. roadside greenery, improvement within parks and at railway stations) supported by Fukuoka City in each ward under the *Flower City Fukuoka* project is listed. This illustrates the number and distribution of smaller-scale actions across the city.

**Table 1:** Population density, green coverage, green space, and number of local greening actions per ward.

Ward	Population density (people per km^2^) (2015)	Ratio of green coverage (2008)	Total area of formal greenspaces (2008)	Area of formal greenspace per person (2008)	Number of local greening actions (Feb 2019)
Higashi	4412	40.7%	4,998,085 m^2^	17.52 m^2^	34
Hakata	7217	25.0%	1,660,490 m^2^	8.08 m^2^	75
Chuo	12,512	22.2%	1,688,954 m^2^	9.70 m^2^	145
Minami	8259	36.7%	1,274,066 m^2^	5.16 m^2^	26
Jonan	8191	36.4%	322,280 m^2^	2.50 m^2^	25
Sawara	2273	79.3%	669,892 m^2^	3.17 m^2^	26
Nishi	2459	67.8%	1,883,301 m^2^	10.16 m^2^	20

Data sources: Fukuoka City Statistical Bulletin 2 (2016b); Fukuoka City, 2009c, Fukuoka City, 2009d; Fukuoka City Flower City Fukuoka (2019).

Given the focus of this profile on climate adaptation, it is worth noting recent experience with natural disasters, weather and climate in the city. Whilst not as seismically active as other areas in Japan, Fukuoka was struck by a powerful earthquake registering *shindo* 6 (the most powerful being *shindo* 7) on March 20,2005, causing damage to buildings, killing one person and forcing evacuation of much of Genkai Island at the edge of Hakata Bay. In 2009, heavy rainfall in northern Kyushu in late July caused increases in river water levels. This resulted in flooding in many parts of Fukuoka, forcing citizens to temporarily evacuate and causing five deaths in the Fukuoka metropolitan area (Hashimoto & Saito, 2012). The summer 2018 Japanese heatwave affected Fukuoka too. A temperature of 38.3 degrees Celsius was observed in Fukuoka City on 20 July 2018, the highest since records began in 1890 (Japan Meteorological Agency, 2019). A record 821 people in the city were hospitalised by heat stroke over the summer of 2018 (up to 30 September), an increase of 241 people over the same period in 2017 (Fukuoka City, 2018b). Based on observational research into Fukuoka's thermal environment, the Hakata-Tenjin area was identified as a ‘hot spot,’ and has subsequently been the focus of further research and experimental measures to mitigate heat island effects (Fukuoka City, 2016a; Tanaka, Hagishima, Kitayama, & Yoda, 2009). Table 1 shows that community and private sector greening activities, whilst not undertaken explicitly for heat mitigation, are also focused on the Hakata and Chuo Wards (where Hakata-Tenjin is located) where green coverage is lower. Flood risk and evacuation maps have been produced for each ward, identifying areas at risk in the event of inundation and illustrating evacuation routes and shelters (Fukuoka City, 2011).

## 6 Focus: climate change adaptation in Fukuoka through the built environment

Compared to Japan as a whole, Fukuoka has experienced above-average warming to date and is likely to continue to feel the effects of climate change into the future. This is reflected in the risks identified in the city's climate change planning and the measures proposed in response. Average annual air temperatures in Fukuoka Prefecture increased by 2.54 °C between 1898 and 2017, compared to 1.69 °C for the wider Kyushu and Yamaguchi area and 1.19 °C for Japan over the same period (Fukuoka District Meteorological Observatory, 2017). Under the IPCC's A1B Emissions Scenario (very rapid economic growth, global population peaking in mid-century and declining thereafter, and rapid introduction of new and more efficient technologies with a balance of fossil- and non-fossil sources (IPCC, n.d.)), average air temperatures in Fukuoka Prefecture are predicted to increase a further 2.9 °C by 2100, with 18 more extremely hot days (over 35 °C) and 42 more hot days (over 30 °C) per year predicted by 2100 (Fukuoka District Meteorological Observatory, 2014).

Fukuoka City's Climate Change Countermeasures Action Plan accordingly identifies five key climate risks requiring adaptation actions: (a) natural hazards from heavy rainfall and flooding; (b) pressure on water resources; (c) health risks from increased heat; (d) biodiversity loss; and (e) effects on agricultural produce (Fukuoka City, 2016a). Heavy rainfall leading to flooding in Fukuoka City in July 2009, and the significant increase in consecutive hot days observed in summer 2013 within Fukuoka are cited as examples of current climate effects (Fukuoka City, 2016a; Japan Meteorological Agency, 2013).

### 6.1 Putting Fukuoka in context: cities and climate change adaptation in Japan

Within the wider Japanese context, Fukuoka may be considered one of the relatively early adopters of climate change thinking in urban planning. The actions proposed in Fukuoka in response to climate risks are perhaps not unique to the city, but what is distinctive is the comparatively high degree of evidence and specificity provided by the city's greenspace and urban plans.

Local governments in Japan generally started to form climate change plans from the mid-1990s onwards, to support national obligations under the Kyoto Protocol. These activities were also supported by the 1993 Basic Environment Law, which integrated wider environmental considerations into previous legislation focusing on anti-pollution measures. The production of climate change plans has been a legal obligation for larger local governments – including Fukuoka City – since 2008 due to the Act on Promotion of Global Warming Countermeasures. From 2018, local governments are obliged to develop plans specific to adaptation under the Climate Change Adaptation Act, and also to nominate a local adaptation centre for data collection and provision. As Table 2 shows, it is hence not unusual for larger local governments in Japan to have local climate plans preceding national legislation. It is however notable that Fukuoka's first climate change plan came earlier than other large cities in west Japan (in addition to those listed in Table 2, Kyoto's plan came in 1997 and Osaka's in 2002) and indeed pre-dated the Kyoto Protocol.

**Table 2:** Comparison of climate adaptation-related actions for Fukuoka, Kobe and Hiroshima.

	Fukuoka	Kobe	Hiroshima
Population (January 2019)	1,582,368	1,526,639	1,199,543
Climate classification	Humid subtropical	Humid subtropical	Humid subtropical
First municipal climate change plan (as stated by city itself within current policy)	1994	2000	2003
Most recent municipal climate change plan	2016	2015	2017
Adaptation issues and countermeasures in most recent municipal climate plan	**Flooding/heavy rainfall** (engineering and river management, preparation of overflow reservoirs, public flood hazard maps and apps); **pressure on water resources** (water conservation advice for public, improve water provision infrastructure); **heat risk** (heatstroke advice for public, online real-time heat risk information, promote roof- and wall greening as part of wider greening and utilise wind corridors, water scattering, provision of cool shelters); **biodiversity loss** (preserve existing habitat, alien invasive species countermeasures, surveying); **effects on agricultural produce** (introduce new climate-resistant produce, changes to farming practices, information provision on habitat effects) (Fukuoka City, 2016a).	**Heat risk** (heat alerts and heatstroke advice for public via events, leaflets, posters and announcements; aim for green network and wind corridors**); spread of infectious diseases via insects, water and plants** (monitoring; risk communication via leaflets); **effects on farming and fisheries** (introduction of new species types, monitoring); **flooding** (river management, online rainfall maps); **storm surges** (sea defences and pumps); **landslides** (engineering, risk communication); **biodiversity loss** (species mapping with citizen observation; surveying; habitat protection) (Kobe City, 2015).	**Debris flows and landslides**; **heat risk** (heatstroke advice for public, aim for green network and wind corridors, promote roof- and wall greening, water scattering, provision of cool shelters); **flooding**; **spread of infectious diseases**; **infrastructural damage**. Stated that many climate risks considered within wider disaster prevention measures. Societal engagement for all climate risks: information on city homepage; create books for schools; hold symposia and seminars; train staff responsible for engagement. Promote collaboration with national government and universities to understand future effects (Hiroshima City, 2017).
Consideration of climate adaptation in most recent greenspace- and urban plans	**Greenspace Plan (**Fukuoka City, 2009c, Fukuoka City, 2009d**)** Mention of specific areas and actions (ACROS Fukuoka, greening of buildings) to mitigate heat island effects; Specific action point to mitigate heat island effect through urban greening in city centre. Supported by thermal images of specific locations (e.g. Tenjin Central Park), temperature data for other locations (e.g. Ohori Park), and map showing relationship between green ratio and temperature at neighbourhood level (data from research led by city government); Focused heat island mitigation action for Hakata-Tenjin core area, identifying wind corridors, ‘cool spots’, areas for greenery preservation, and buildings due for renewal; Survey of citizens shows urban heat mitigation second-biggest perceived value from greening; Mention of need to create/preserve wind corridors, with specific identification: Naka River, Watanabe Road, Taihaku Road; Statement of increase in green ratio for purpose of heat island mitigation; Encouragement of wall- and rooftop greening to mitigate heat island effects. **Urban Plan (****Fukuoka City, 2014****)** Mention of aim to reverse declining trend of greenery in city and develop green network to realise climate and heat mitigation benefits; Preservation of wind corridors from Hakata Bay, image of greenery in Tenjin Central Park; Provision of pumps and storage reservoirs for intensifying rainfall.	**Greenspace Plan (****Kobe City, 2011a****)** Mention of risk to lifestyles and livelihoods from biodiversity loss due to climate change; Survey of citizens shows urban heat mitigation biggest perceived value from greening; Land acquisition and tree planting for purpose of climate response; Plan to create wind/green corridor and associated green network from sea to Mt. Rokko to aid cooling. **Urban Plan (****Kobe City, 2011b****)** Inclusion of summer wind/temperature map produced by Kobe University, with explanation of value of creating wind corridor for cooling; Plan to create wind/green corridor and associated green network from sea to Mt. Rokko to aid cooling; Provision of wind corridor listed as benefit of actions (tree provision, protection of farmland, satoyama practice) across city; Pumping and storage ponds for heavy rainfall.	**Greenspace Plan (****Hiroshima City, 2011****)** Mention of need for heat island mitigation; City divided into 5 zones, with different greening actions in response to climate change listed for each: Island (preserve forests); Delta City (protect private greenery, including small-scale; encourage greening of roads, factories, and green walls and roofs); Aogaki mountain area (protect mountain forests to connect with Delta City greening and create a green infrastructure); New Town (conserve belts and islands of forest in urban areas; protect mountain forests during urban development); Inland Area (protect mountain forests including river banks). **Urban Plan (****Hiroshima City, 2013****)** Mention of need for response to rainfall intensification and associated flood risk; Mention of generic climate response benefits from biodiversity and environmental protection as part of parks and greenspace; Mention of heat island mitigation through greening: public space (schools, roads parks), citizen/private greening, forests; also protection of wind corridors (computer visualisation of street trees and green wall in Naka Ward).

Table 2 compares Fukuoka's actions on climate change within greenspace- and urban planning to two other western Japanese coastal cities of similar size and climatic characteristics – Hiroshima and Kobe. The intention is simply to provide some comparative context, and not to compare or criticise the cities in question. However, it is striking that Fukuoka's greenspace plan in particular contains a relatively high level of detail and specificity, especially for urban heat island mitigation.

### 6.2 Policy landscape for climate adaptation in Fukuoka

Table 3 summarises the main policies informing climate adaptation via planning in Fukuoka City. The core guiding policy is the Fukuoka City Climate Change Countermeasures Action Plan (2016a), known as the *Cool and Adapt Project* for short. This was developed to integrate the city's local climate change plan and its broader environmental plan. Previously, Fukuoka City Government produced its first local climate change plan – the Fukuoka City Local Climate Change Countermeasures Promotion Plan - in 1994, which was updated in 2001 and again in 2006. Climate issues are also considered within greenspace planning in Fukuoka, via the Fukuoka City, 2009c, Fukuoka City, 2009d. This replaces the Fukuoka City Green Basic Plan (1999), and is due to be updated by 2020. The Fukuoka City Urban Planning Masterplan (2014) also discusses climate issues, albeit largely in relation to mitigation and with adaptation actions similar to those in the New Green Basic Plan.

**Table 3:** Main policies informing climate adaptation in Fukuoka City.

Policy	Year introduced	Responsible organisation	Identified areas requiring adaptation action	Proposed adaptation actions relevant to Fukuoka City
Fukuoka City Climate Change Countermeasures Action Plan	2016	Fukuoka City Environment Division	Flooding/heavy rainfall; pressure on water resources; health risk from heat hazard; biodiversity loss; effects on agricultural produce.	Hazard mapping and public communication; developing emergency reservoirs; protection of greenspaces and rivers; promotion of behaviour change.
Fukuoka City New Green Basic Plan	2009	Fukuoka City Housing Division, Green City Promotion Section	Flooding/heavy rainfall; heat island effects.	Preservation of wind corridors; connecting mountains and sea via ‘green corridors’; creation of ‘green network’; development of green areas for stormwater retention; focus on special measures in Tenjin-Hakata Station ‘hot area’.
Fukuoka City Urban Planning Masterplan	2014	Fukuoka City Housing Division, Urban Planning Section	Flooding/heavy rainfall; heat island effects.	Preservation of greenspaces and rivers; creation of ‘green network’; developing emergency reservoirs (as per New Green Basic Plan).
Fukuoka Prefecture Climate Change Countermeasures Action Plan	2017	Fukuoka Prefecture Environment Division, Environmental Protection Section	Flooding/landslides from heavy/intense rainfall; health risks from heat hazard; effects on agricultural produce.	Understand green infrastructure and ecosystem service potential in adaptation via research; behaviour and practice change among agricultural workers; hazard mapping and public communication; stormwater management.
National Plan for Adaptation to the Impacts of Climate Change	2015	Government of Japan	Reduction in crop quality, drought; species extinction; flooding/landslides from increased rainfall; increases in storm surge and high wave risk; increased summer heat waves.	Promotion of development of local adaptation plans; provision and development of local-level climate data and locally-specific information; support model projects and knowledge-sharing.
Climate Change Adaptation Act	2018	Government of Japan	7 priority areas: agriculture, forestry, fisheries; water environment and resources; natural ecosystems; natural disasters; human health; industries and economic activity; life of citizens.	Municipalities mandated to form Local Climate Change Adaptation Plans, and also to assign a Climate Change Adaptation Center for data collection and provision.

At regional level, the Fukuoka Prefecture Climate Change Countermeasures Action Plan (2017) provides climate change response guidance for all of Fukuoka Prefecture (including rural areas and Kitakyushu City as well as the Fukuoka urban area), setting guidelines which are enacted via Fukuoka City. As outlined previously, these actions take place under national-level legislation set by the Japanese Government: Act on Promotion of Global Warming Countermeasures (2008); National Plan for Adaptation to the Impacts of Climate Change (2015); and Climate Change Adaptation Act (2018).

### 6.3 Evidence base for climate adaptation via planning in Fukuoka

As outlined in Section 6.1 and Table 2, what is notable is that whilst Fukuoka is one of a group of cities which were early to engage with climate issues in urban governance, the city's plans appear grounded in a comparatively rich techno-scientific evidence base. Fukuoka has a history of environmental science research and planning considerations aimed at understanding how the built environment may be configured to create a comfortable urban climate (Mabon et al., 2019). This predates the city's current interest in climate change adaptation.

Understanding of the urban climate in Fukuoka City – in particular the thermal environment - is underpinned by evidence from both national and local levels. Under the direction of the Ministry of Land, Transport and Infrastructure, Fukuoka was one of the first two municipalities in Japan – along with Tokyo – to produce an urban climate atlas, creating a climate function map focusing on effects of green space on urban heat islands with urban climate simulation system (Ichinose, Mikami, Niitsu, & Okada, 2003). Remote sensing-based studies aimed at understanding how planning can mitigate heat effects have been conducted in Fukuoka by researchers at local institutions since at least the early 1990s (e.g. Katayama et al., 1990), with Fukuoka City Government undertaking a neighbourhood-level study into thermal environments in 2006 in collaboration with locally-based researchers (e.g. Tanaka et al., 2009).

Academic knowledge and research findings connect with local government policy through the participation of researchers from local institutions (e.g. Kyushu University/Kyushu Institute of Design, Fukuoka University, Kyushu Environmental Evaluation Association/Fukuoka Center for Climate Change Actions) on expert committees for the adaptation-related plans listed in Section 6.1. This provides a contrast to Baba et al. (2017), who identify a lack of expert knowledge and experience at the municipal level as a barrier to local climate adaptation in Japan. The role of Naohito Asano of Fukuoka University at the academia-policy interface is particularly noteworthy. Asano was an expert panel member for Fukuoka City's *Cool and Adapt Project*, and also chaired the expert committee for Fukuoka Prefecture's Climate Change Countermeasures Action Plan. What is significant is that Asano is an environmental lawyer whose career has been conducted largely in Fukuoka, with a professional interest in the use of local urban planning legislation and policy as a way to secure quality of living for citizens (e.g. Asano, 1988). As such, the connection of the research base with planning policy actions has been aided by a community of academics with an understanding of the technical and scientific principles of urban climate adaptation gained at many cases at local institutions, *and* also knowledge of the legislation and policy that can put these actions into practice.

This rich evidence base may in part be explained by the longer interest in Fukuoka in local environmental quality and in managing the urban climate, both of which pre-date climate adaptation considerations. The incidents of Minamata Disease and air pollution in Kitakyushu both led to calls in the 1960s in Kyushu for independent environmental science undertaken in the public interest (e.g. Fujikura, 2001). The Kyushu Environmental Evaluation Association, which has been involved in monitoring urban thermal environments and in hosting the Fukuoka Center for Climate Change Actions, was specifically set up by academics in the early 1970s to monitor water quality as a result of events in Minamata. Asano too was engaged with the legal aspects of Minamata Disease earlier in his career (Asano, 1992).

**Fig. 5:**
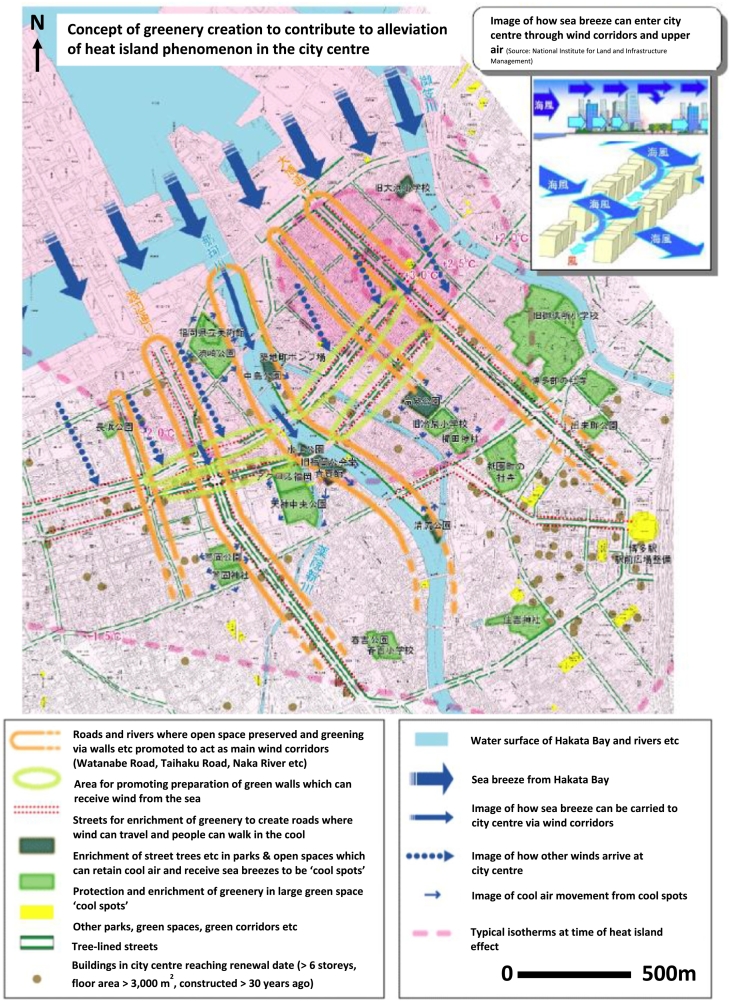
Strategies for preserving/creating wind corridors, creating cool spots via greenspace, and identifying areas of higher temperature included within Fukuoka City New Green Basic Plan.

There is also a concurrent interest in Fukuoka in the provision of *kaiteki kankyou* – a liveable environment – through urban planning and environmental governance. This language was utilised in Fukuoka City's first Environmental Plan (Fukuoka City, 1986), and also in the writings of Fukuoka-based academics working at that time (e.g. Asano, 1988; Mitsuyoshi, 1985). A group of academics from Kyushu University of Design (now part of Kyushu University) in 1981 published *Design of micro-weather in urban greening*, which assessed how urban greenspace at a neighbourhood- and city-scale could be used to ensure thermal comfort (Nitta et al., 1981), tallying with Hebbert's (2014) observation that Fukuoka was one of the Japanese cities to engage early with ideas of urban climatological planning. Indeed, the Fukuoka City Green Basic Plan (1999) and subsequently the Fukuoka City, 2009c, Fukuoka City, 2009d consider such ideas of preserving wind corridors, creating a city-wide green network, and targeted strategic greening for cooling (see Fig. 5).

(Source: Fukuoka City, 2009c)

Climate adaptation actions via planning in Fukuoka are thus supported by a number of local institutions with significant competence in understanding the urban environment, and willingness to engage with the policy-making process. However, this is arguably the result of a much longer concern in Fukuoka with a liveable local environment, which has helped these local institutions to develop the skills to create an environmental science evidence base towards maintaining quality of life (Mabon et al., 2019). This appears particularly true when it comes to mitigating heat effects. Whilst Hijioka et al. (2016) are right to claim that Japanese cities already have a number of robust local environmental and social policies which give a basis for climate change adaptation, it may hence also be the case that Fukuoka's distinctive environmental history has supported the comparatively early and rigorous consideration of climate adaptation concerns within Japan.

### 6.4 Urban environment climate adaptation successes to date

As above, ‘success’ may come through Fukuoka's early adoption of climate change and adaptation-related policies, especially the specificity with which these are considered within greenspace and urban plans. In Japan, existing urban policies developed for areas such as disaster risk reduction and environmental management may mean that cities are already relatively well-prepared for climate change adaptation and may not require new areas of policy or planning to deal with weather and climate risks (Hijioka et al., 2016). Indeed, as per Table 2, Hiroshima City Government explicitly states that climate adaptation is considered within wider disaster risk reduction measures. What is perhaps distinctive about Fukuoka is therefore the *explicit* focus on climate change within greenspace- and urban planning measures. Fukuoka City produced its first climate change plan in 1994, placing it ahead of other local governments developing similar plans (e.g. Hiroshima, Kobe, Kyoto, Osaka) by several years. In comparison to other Japanese city contexts, Fukuoka's greenspace planning has also made rigorous attempts to consider climate change, the urban thermal environment and to work towards understanding the city's greenspace in terms of a city-wide green network, supported by research from within the government and by local institutions (Fukuoka City, 1999, Fukuoka City, 2009c). The city's support for research into localised heat island effects, and subsequent utilisation of these findings to inform decisions on development of a grass area outside Fukuoka City Hall (Tanaka et al., 2009), indicates there is a willingness within government to consider urban climate issues within their governance of the built environment. Moreover, although at an early stage, it is significant that Fukuoka Prefecture's Climate Change Countermeasures Action Plan (2017) identifies the need to understand green infrastructure and ecosystem services across the Fukuoka area from now into the future.

**Fig. 6:**
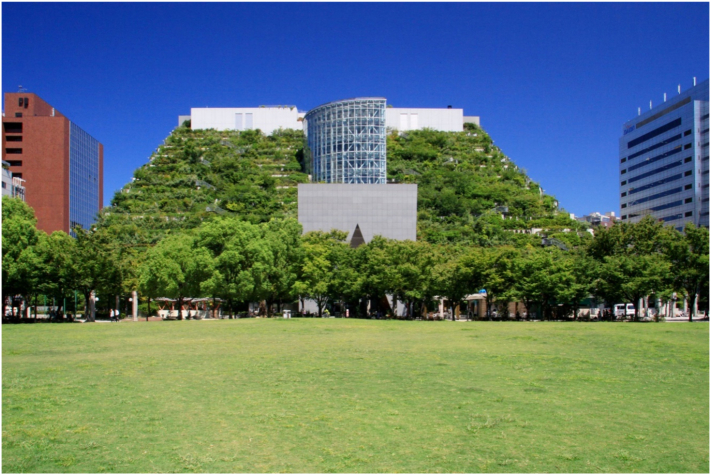
ACROS Fukuoka.

**Fig. 7:**
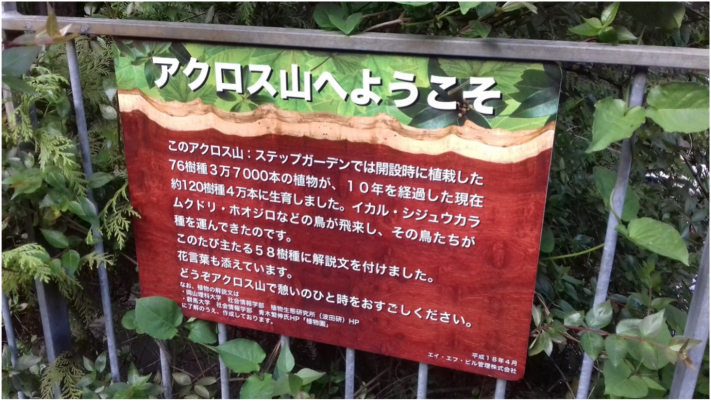
Information about biodiversity at entrance to ACROS Fukuoka.

The ACROS Fukuoka Prefectural International Hall is an example of a location where greening has delivered local cooling and also biodiversity benefits (see Fig. 6, Fig. 7). Completed in 1995 and designed by green architecture pioneer Emilio Ambasz, ACROS features woodland on the stepped façade of a 15-storey building (Jim, 2017a) and is directly connected to the adjacent Tenjin Central Park. The building provides public good by containing a concert hall and government service points, as well as restaurants and office space. It is reported to host 40,000 plants and trees spanning 120 species. The ACROS terraces were not designed to provide climate or biodiversity benefits. Rather, the focus was on aesthetics, and on reducing the general environmental impact of the development (Jim, 2017a). However, the terraces have been demonstrated to provide rainfall runoff quality benefits (Berndtsson, Bengtsson, & Jinno, 2009); and to deliver cooling effects in the immediate area (Hagishima, 2018). Internationally, ACROS is a well-known example of vertical greening. ACROS was featured in an article on flood risk reduction from green roofs in academic blog site *The Conversation* (Howell, Drake, & Margolis, 2017), and included in an article on green roofs and heat island mitigation in UK newspaper *The Guardian*'s Cities section (The Guardian, 2018). Both in academic literature and also public-facing material aimed at informed audiences, then, ACROS appears to be viewed internationally as a success story of climate change adaptation.

(Source: authors' own photo)

(Source: authors' own photo)

Locally, signage on the terraces explains ACROS' biodiversity (authors' own visit, 2018), and the image of ACROS is frequently used in public-facing material relating to urban climate in Fukuoka (e.g. the New Green Basic Plan Summary Leaflet, (Fukuoka City, 2009d)). The site hence has a symbolic role within Fukuoka in motivating action towards climate change countermeasures and urban climate protection. Since ACROS was built, other large-scale green roofs have developed in Fukuoka. The *Grin Grin* active learning space and botanical garden in Island City Central Park has a green roof area of 1000 m^2^ over three connected buildings and opened in 2005 (Island City Central Park, 2019). The *Tenjin Core* shopping centre also installed a green roof, but is scheduled to close as part of the Tenjin Big Bang intensification works. Fukuoka City Government is responsible for the *Green Curtain Project* and *Flower City Fukuoka* initiatives, which collaborate with citizens and the private sector to encourage development of green walls and localised greenery respectively.

Indeed, another aspect in which Fukuoka has made progress in climate adaptation through the built environment is in community and citizen engagement in greening. Engagement of communities in greening strategies has been encouraged to maximise climate benefit from greening in dense cities where greenspace may be at a premium and available space may lie outside formal greenspace plans (Mabon & Shih, 2018; Tan & Jim, 2017). Engagement on community-level greening can also help to build social relations (Tidball & Atkipis, 2018) and hence perhaps increase resilience to future climate events. As part of the *Green Curtain Project*, Fukuoka City offers cash prizes for growing vertical greenery (Fukuoka City, 2016a). The competition is framed in terms of delivering cooling and reducing energy consumption, and is supported with training sessions in growing and maintaining greenery. Fukuoka City Botanical Gardens also operates a ‘Green Advice Centre’ providing free technical advice to citizens and community groups wishing to develop greenery in their locale. Whilst not specifically targeted at climate adaptation or maximisation of ecosystem services, this nevertheless goes some way to addressing the lack of technical competence (Jim, 2017b) which may act as a barrier to enhancing or maintaining urban greenery within a city.

### 6.5 Challenges

Thus far, this profile has illustrated that Fukuoka has a sound policy base for climate adaptation action through competences in greenspace and urban planning in response to environmental change. However, the extent to which this basis extends to other urban policy areas, or to tangible action, may be more limited.

The first difficulty lies in putting the visions laid out in the city's greenspace plan fully into action. Whilst there is very good knowledge stretching over three decades of the urban climate in Fukuoka and of how it may be controlled via greening (e.g. Hagishima, 2018; Nitta et al., 1981; Yoda & Katayama, 1998), interventions in the built environment are perhaps limited beyond the flagship examples laid out in Section 6.4. For more expansive measures such as the preservation or construction of wind corridors, it is also very difficult if not impossible to remove or alter existing buildings along the seafront (interview with KEEA, April 2018). The data presented in Section 5.2 indicates that Fukuoka's continuing growth is putting pressure on green spaces, with a declining trend in green coverage at the time of the last greenspace planning cycle. Nonetheless, this is recognised by the city, and the inclusion of more green and open spaces within the city's formal greenspace system – as well as city government-led initiatives such as the *Green Curtain Project* and *Flower City Fukuoka* – indicate efforts to re-establish greening as part of adaptation efforts.

The second challenge represents the observations of Baba et al. (2017) that compartmentalisation of government bureaus may be a barrier to local climate adaptation progression in Japan. Whilst the Fukuoka City Urban Planning Masterplan and the Fukuoka City New Green Basic Plan both come under the remit of the Housing Division, the Climate Change Countermeasures Action Plan falls under the remit of the Environment Division. These local government divisions themselves report to different sections of national government. Housing is connected to the Ministry of Land, Infrastructure and Transport; and Environment to Ministry of Environment. Interviewed local government officials acknowledged there are initiatives to link different areas of local government through periodical face-to-face forums and also the connection of policies and plans to each other, but also that greenspace and climate adaptation plans do to an extent develop in silos (interview with Fukuoka City Environment Division, May 2017; interview with Fukuoka City Government Green City Promotion Department, April 2018). At the prefectural level, the Fukuoka Prefecture Climate Change Countermeasures Action Plan (2017) does however assign the identified tasks relating to climate change to specific prefectural government sections and departments. This may represent a move towards the “integrated adaptation strategy for the whole local government” which Baba et al. (2017: 10) believe is missing in Japan at present.

Third, there is a potential tension in Fukuoka between economic development and environmental protection. Fukuoka has the highest growth rate of local tax revenue (4.2% between 2008 and 2015) of all major cities in Japan, and the fastest-growing population of major Japanese cities (7.1% between 2010 and 2017) (Fukuoka Asian Urban Research Center, 2018). Partly in response to this growth and associated demand for office and hotel space, Fukuoka City Government has initiated the ‘Tenjin Big Bang’ project described in Section 5. The Tenjin Big Bang regulations do incentivise pro-environmental actions in redevelopment by granting an increase in floor-area ratio for reduction of environmental impact (Fukuoka City Government, 2018c). This may help to encourage greening, given that Fukuoka City Government has tried, so far without success, to include greening actions within building codes due to perceived conflict with economic development (interview with Fukuoka City Government Green City Promotion Department, April 2018). However, although there is no clear opposition in Fukuoka yet, similar experiences in Taipei indicate that if not managed sensitively, such initiatives to engage developers in urban greening initiatives can backfire and/or be met with scepticism by pro-environmental and civil society stakeholders if they are perceived as giving developers too much power in planning processes (Mabon & Shih, 2018).

There are also challenges in Fukuoka which reflect the general adaptation situation for Japanese cities. These include the still strong focus on mitigation compared to adaptation in the *Cool and Adapt Project*, which tallies with Kameyama's (2016) general observation on Japanese municipal climate policy. Respondents also commented (interview with KEEA, April 2018) that the conditions of government-funded projects sometimes made data sharing or re-use for further research into local climate adaptation difficult, an observation also made for Tokyo by Hijioka et al. (2016).

## 7 Conclusion

This City Profile has illustrated that within Japan, Fukuoka has made relatively early progress on local governance of climate change adaptation, particular as regards consideration of specific implementation actions when compared to cities in western Japan of similar size and climate. What is perhaps most distinctive is that there is in Fukuoka a robust body of scientific evidence produced by local scholars and institutions on which municipal policymakers may draw to support their decisions. Our profile also supports the argument that whilst climate change adaptation may be a relatively new area for local governments in Japan, there are already comprehensive policy frameworks in place in areas such as environmental protection, disaster prevention, and appropriately-informed greenspace planning which can act as a foundation to facilitate actions necessary for climate adaptation (Hijioka et al., 2016; Morimoto, 2011). In Fukuoka, greenspace planning policies appear particularly well-suited to adapting to the effects of increased heat and rainfall, in that explicit consideration of climate adaptation may be considered only the latest iteration in the city of a much longer policy and research interest into how the urban green environment can be configured to create a ‘liveable environment’ for citizens.

Nonetheless, there are limitations to the applicability of Fukuoka's experience with climate adaptation to other international cities. Whilst Fukuoka is indeed an expanding, subtropical, medium-sized city in Asia, the current and historical context makes it a distinctive and in some ways anomalous case. The strong technical and scientific competences in understanding the local environment held by local institutions and researchers in Fukuoka, and the associated drive to connect this with policy-making and undertake research in the public interest, may to an extent be the result of Kyushu's specific historical experiences with pollution. Fukuoka is also a large, well-resourced and still-growing city in a wealthy country context. This may afford the city access to funds to support the development of comprehensive climate and environmental policies, and also aid the recruitment and training of technically capable local government staff. Whether such actions would be possible to the same extent in other rapidly developing subtropical Asian cities without this level of resourcing and the specific social and environmental history is open to question.

## Declaration of Competing Interest

Hiroyuki Kanekiyo was a member of the Academic Expert Committee for the Fukuoka City Urban Planning Masterplan (2014). However, no funder or organisation has had any influence over the content of this City Profile, or over the research on which it is based.

## Footnotes

1 We use ‘local’ to represent policies and actions undertaken at the municipal (i.e. city) level, and ‘regional’ to represent the prefectural level.
